# Generation of Mixed-OAM-Carrying Waves Using Huygens’ Metasurface for Mm-Wave Applications

**DOI:** 10.3390/s23052590

**Published:** 2023-02-26

**Authors:** Hassan Naseri, Peyman PourMohammadi, Nouredddine Melouki, Fahad Ahmed, Amjad Iqbal, Tayeb A. Denidni

**Affiliations:** Centre-Energie Matériaux et Télécommunications, Institut National de la Recherche Scientifique, Montréal, QC H5A 1K6, Canada

**Keywords:** orbital angular momentum (OAM), antennas, Huygens’ metasurface, transmit array (TA), Mm-wave applications

## Abstract

Antennas that generate orbital angular momentum (OAM) have the potential to significantly enhance the channel capacity of upcoming wireless systems. This is because different OAM modes that are excited from a shared aperture are orthogonal, which means that each mode can carry a distinct stream of data. As a result, it is possible to transmit multiple data streams at the same time and frequency using a single OAM antenna system. To achieve this, there is a need to develop antennas that can create several OAM modes. This study employs an ultrathin dual-polarized Huygens’ metasurface to design a transmit array (TA) that can generate mixed-OAM modes. Two concentrically-embedded TAs are used to excite the desired modes by achieving the required phase difference according to the coordinate position of each unit cell. The prototype of the TA, which operates at 28 GHz and has a size of 11 × 11 cm ${}^{2}$, generates mixed OAM modes of −1 and −2 using dual-band Huygens’ metasurfaces. To the best of the authors’ knowledge, this is the first time that such a low-profile and dual-polarized OAM carrying mixed vortex beams has been designed using TAs. The maximum gain of the structure is 16 dBi.

## 1. Introduction

The next generation of wireless systems require high rates of data, and the existing antennas cannot satisfy this demand due to limited bandwidth (BW) and signal to noise ratio (SNR). Thus, another degree of freedom should provide users with their expected communication connections. OAM antennas are good candidates to solve this issue [1,2]. These kinds of antennas, unlike current ones, carry the signals in their phase front. In other words, each specific mode of an OAM antenna has a helical phase front which is orthogonal to other modes’, and as orthogonal phase fronts are independent of each other, they can be responsible for transmitting their own data streams. However, in traditional antennas, to transmit multiple signals using an antenna, the BW should be divided into several sections, resulting in a lower channel capacity and consequently low data rate. In this regard, to take advantage of the characteristics of OAM antennas, multiple modes must be generated from one aperture.

Referring to the literature, many structures capable of generating OAM modes can be found. These structures can be categorized into four groups: antennas having one input port providing reconfigurable OAM modes, antennas generating multiple OAM modes by having an individual port for each mode, single antenna elements, and metamaterials. For the first case, in [3], a 2 × 2 array including circular patches was utilized to bring about a uniform circular array for exciting tunable OAM modes +1 and −1. The same patch antennas with a different feeding network were used to create the same tunable modes in [4]. The authors of [5] take advantage of changing the direction of input signals to alter the phase value of equally divided signals feeding the antenna elements, giving rise to the generation of reconfigurable modes. Another phase-shifting reconfigurable antenna feed introduces a system with both tunable OAM modes and polarization agility [6]. Using a circular patch antenna with two arc segments in the connection between the feed and the resonator, second-order OAM modes are generated in [7]. A wideband single fed circular polarization array enables the propagation of reconfigurable dual-mode OAM beams [8]. OAM modes −1, 0, +1 are excited by means of electrically mode-tunable metasurface antenna [9]. The authors of [10] introduce a simplified feeding network to generate multiple OAM modes in six tunable states. A 1-bit reconfigurable reflectarray provides the condition for generating mode switching OAM waves in [11]. A similar work, this time using a transmit array, is proposed in [12]. In [13], vortex beams showing circular polarization are excited using a mechanically reconfigurable antenna array. Spoof surface plasmon ring resonators pave the way for the creation of adjustable-OAM modes in [14]. A water antenna in [15] was designed to propagate OAM-carrying beams with the aid of a reconfigurable feeding network. It should be noted that in most of the aforementioned references, PIN diodes were used while designing the reconfigurable feeds. However, very recently, Hassan et al. have presented a low-profile structure which can enable the excitation of tunable OAM modes using Varactor diodes [16]. This leads to solutions to the problems associated with the leakage of signal in the reverse bias state of PIN diodes.

Regarding the structures having individual ports for each mode, in [17], two separate feeding networks produce phase responses of [0, 90, 180, 270] and [0, −90, 0, −90] degree for modes +1 and −1, respectively. In [18], four OAM modes are engendered using four different antenna feeds. OAM modes −1 and −2 are excited using two different feeding networks plated on the same layer. OAM mode −3 has its own feed on one layer, and OAM mode 0 is created by a centered patch antenna. Sequentially rotated configuration of the antenna elements in a uniform circular array paves the way for the simplification of the feeding networks. This feature was considered in [19] in order to obtain modes +1 and +2 with low-profile antenna feeds. However, achieving the mentioned modes without using the sequential rotation method would lead to designing complicated and lossy feeds. Radial Line Slot Arrays (RLSA) can also be utilized in engendering OAM-carrying waves [20]; a 4 × 4 Butler Matrix (BM) provides the condition for OAM modes of 0, −1 and +1 to be propagated. A quad-mode uniform circular array was suggested in [21]. In this structure, the arrays are fed by hybrid couplers connected to ring-shaped transmission lines. Some other examples of structures having multiple input ports for multiple OAM mode generations can be found in [22,23,24,25,26].

Besides the structures belonging to the two categories already mentioned, reconfigurable OAM antennas and those having individual ports for each mode, there exist a few single elements which can excite OAM modes as well. However, these structures have low gains in comparison with the works using arrays. Among the single antenna elements producing low-gain OAM modes, the structures proposed in [27,28,29,30,31,32,33,34] can be taken into consideration. In [27], a circular patch antenna is fed using two hybrid couplers to provide OAM modes with high purities. It should be mentioned that the OAM modes are excited in the second resonance frequency of the patch. A square patch antenna is fed from its four sides to create OAM modes in [28]. The authors of [29] proposed an octagonal structure to excite higher-order OAM modes. A substrate integrated waveguide (SIW) cavity is utilized in its half mode [30] to bring about vortex beams. A traveling-wave ring-slot structure is responsible for enabling the propagation of four OAM-carrying waves in [31]. A cylindrical dielectric resonator antenna can generate several OAM beams at different frequencies [32]. Finally, a water-immersed rectangular horn antenna and a spiral antenna can result in vortex beam generation, as in [33,34], respectively. To come up with a high-gain single element structure, leaky wave antennas have also been designed [35,36]. Although both low- and high-gain single element antennas are appropriate components for compact wireless systems, they suffer from the possibility of generating more than two OAM modes at a specific frequency.

Metamaterials have been used for various applications, including filter-antennas sensors [37], all-metal metamaterials for high-gain applications [38], beam-steerable frequency selective surface (FSS)-based phase shifting surfaces [39], ultra wide band (UWB) and wide band FSS-based antennas and filters [40,41]. Several works related to metamaterial-based OAM antennas exist in the literature. Among them it is worth referring to [42,43,44,45,46,47,48,49,50]. It should be noted that a full review of guided-wave metasurfaces for vortex beam generation has been reported in [51]. The metamaterial-based structures mentioned can create only one OAM beam, resulting in them being inappropriate for communications with high data rates. One way to achieve the generation of various modes is to use mixed-OAM-generating antennas. Mixed-OAM modes, which can be used for transmitting several versions of a data stream as MIMO antennas can, are barely achieved using single port mode reconfigurable structures. In the case of those structures that have individual ports, the design complexity is a barrier for obtaining high-gain antenna systems. Furthermore, it is a difficult task to design antenna feeds for the latter cases. To resolve these issues, a few works have been reported which enable the excitation of mixed-OAM modes with the aid of TAs and reflectarrays, giving rise to high-gain structures with no need for sophisticated feeding networks [52,53,54,55,56]. The authors of [52] utilized perforated spiral phase plates (SPP), requiring state-of-art equipment for fabrication; [53] considered two layers during the design process, [54] took advantage of cascaded TAs, [55] used two feeds at off-axis positions to superimpose two OAM modes at the broadside in the reflectarray, and [56] carried out the same process by means of a TA.

In this paper, mixed OAM modes of −1 and −2 are achieved using an ultrathin dual-polarized Huygens’ metasurface. The design evolution starts with calculating the angular position of each unit cell in a planar TA. More specifically, the TA should satisfy two conditions; it should change the received spherical wave to a planar one, and alter it to mixed OAM modes −1 and −2 according to the angular position of each unit cell. The measured results confirm the generation of radiation patterns with a null in the broadside and having helical phase fronts. The operating frequency of the TA is considered to be 28 GHz, which is a candidate for 5G networks. In addition, the maximum gains of 16 dBi are measured at both X- and Y-polarization.

## 2. Unit Cell Characteristics

**Figure 1:**
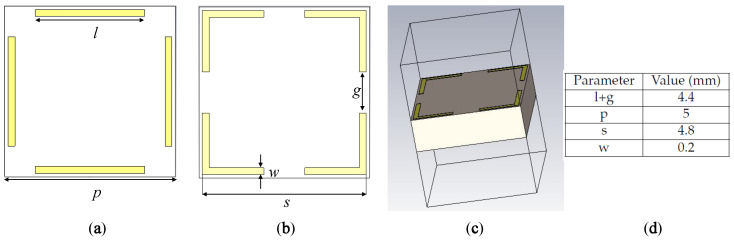
The configuration of the huygens’ metasurface, (**a**) front view, (**b**) back view, (**c**) simulation setup, and (**d**) the parameter values.

An ultrathin Huygens’ metasurface introduced in [57] has the capability of starting the phase shifts from 0 to 360 degrees. Figure 1 shows the configuration of the mentioned unit cell alongside the setup of its simulation carried out in CST software.

The unit cell is printed on both sides of the Rogers 4003C, with a dielectric constant of 3.55 and a thickness of 1.524 mm. Because the unit cell has a square shape and symmetrical profile, it is called dual-polarized. In fact, the polarization of the incident wave does not matter, as a result of the fact that its horizontal and vertical components see the same configuration. It should also be pointed out that during the simulation process, the “unit cell” boundary condition is selected for the plates connected to the unit cell in Figure 1c. For the top and bottom plates, which are in open (add space) boundary conditions, the feeds exist, and by changing the value of *l* and *g* in such a way that *l* + *g* always = 4.4 mm, all the necessary phase compensation ranging from 0 to 360 degrees is achieved. However, for our case, the mixed-OAM-generating TA, only quantized values, which will be discussed later, are taken into consideration. Concerning the distance of the top and bottom boundary boxes from the surface of the unit cell, it should be noted that $\lambda_{0}/2$ was considered, and the reference planes were selected to be on the front and bottom surfaces of the unit cell.

**Figure 2:**
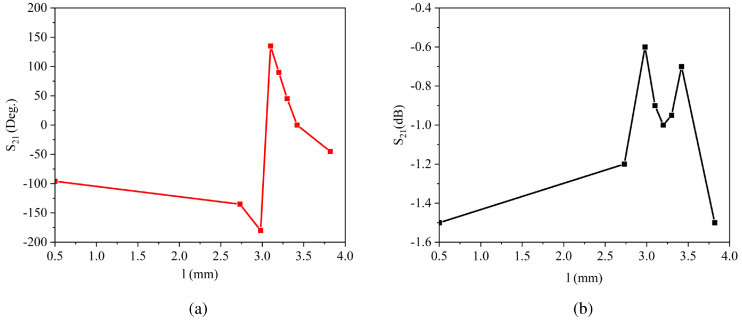
(**a**) Phase response, and (**b**) amplitude response of the unit cell according to different values for *l* (*l* + *g* = 4.4 mm).

Figure 2 presents the variation of the transmitted phase and the amplitude response according to the value of *l*. It is worth mentioning that *l* + *g* = 4.4 mm must be considered in each step. Concerning Figure 2a, the phase response of the unit cell is very smooth once the value of *l* is altered from 0.5 to 2.75 mm. Then, it seems that there exists a sharp change starting from 3 and ending at 3.1 mm. However, this sharp change appears merely because of the periodicity of the phase; subsequently, the phase decreases by increasing the value of *l* up to *l* = 3.82 mm. It should be clarified here that during the parametric study, we only take into consideration the phase values of 0, 45, 90, 135, 180, 225, 270, and 315 degrees. This has its root in the phase quantization technique being used in the generation of OAM modes. When it comes to the amplitude response of the Huygens’ metasurface, it is obvious from Figure 2b that the mentioned unit cell has a low transmission loss and is a perfect candidate for achieving the high-gain OAM waves.

## 3. Mixed-OAM-Generating TA Design

**Figure 3:**
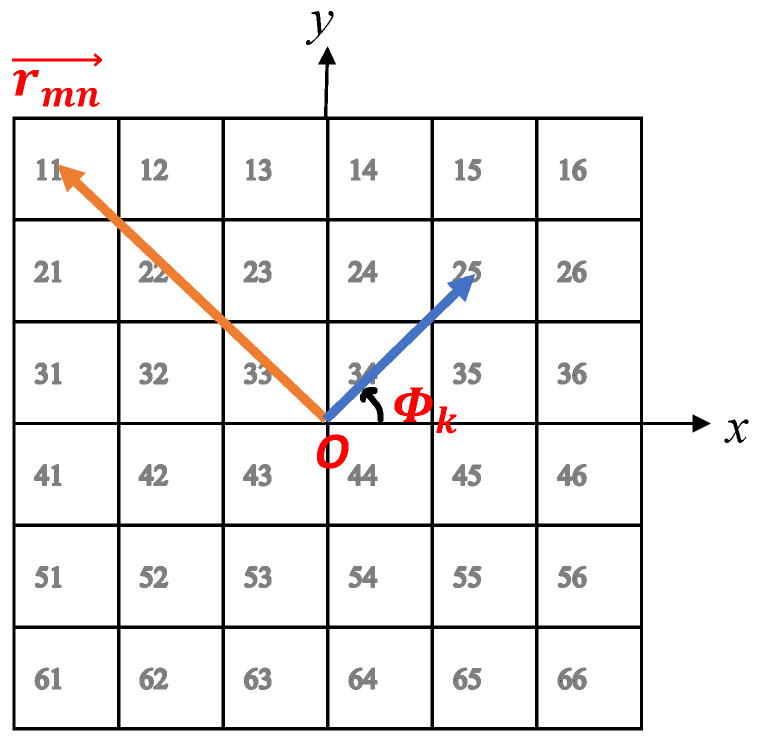
The schematic of a simplified TA including m × n = 6 × 6 elements to demonstrate the variables of Equation (1).

Upon finding the required values for *l* such that the unit cell satisfies the above-mentioned quantized values and has a low transmission loss, the proposed TA can be designed. To that end, two important steps should be taken into account: the TA needs to transform the received spherical wave into a planar one and then create the desired mixed-OAM modes; see Equation (1), which is the summation of the two mentioned steps [58].

(1) $$\phi_{mn}^{c}=-\frac{2\pi }{\lambda }\left|\vec{r_{mn}}-\vec{r_{f}}\right|+arg\left\{e^{\left(j\right)l_{k}.\Phi_{k}}\right\}$$

where $\phi_{mn}^{c}$ is the required phase of the *mn*th element of the TA, which is supposed to have 400 unit cells (m × n = 20 × 20). λ, $\vec{r_{mn}}$, and $\vec{r_{f}}$ are the wavelength at free space considering 28 GHz as the operating frequency, the vector position of each unit cell, and the vector position of the feed antenna (horn), respectively. Further, $l_{k}$ is the OAM mode number, and $\Phi_{k}$ is the azimuth angle of each unit cell in the normal plane of the TA. In order to give a clearer illustration of the components of the equation, Figure 3 may be useful. In this figure, a simplified TA including m × n = 6 × 6 elements is considered.

The first sentence of Equation (1) is related to transforming a spherical wave to a planar wave. In order to achieve a planar wave, all the waves passing through the unit cells that make the TA should become in-phase. In particular, when the wave propagates using a horn antenna as a feed, the electric fields on the TA plane present different phase differences relative to one another. Thus, with regard to the simulated values for *l*, it is possible to make all the fields in-phase.

In the second step, the desired OAM modes are selected, and by paying attention to the second sentence of the equation, the other required phase for each unit cell is calculated. Therefore, the summation of the two phases achieved from the equation provides the precise compensating phase values for one OAM mode. In order to obtain mixed OAM modes, the centered 10 × 10 TA is assigned to mode −1, and the rest of the TA is allotted to mode −2. In other words, from a 20 × 20 TA, the centered 10 × 10 sub-TA generates OAM mode −1 and the remaining part produces mode −2.

After calculating all the necessary compensating phases, it is time to utilize quantized values according to Equation (2).

(2) $$\phi_{mn,quantized}^{c}=\left\{\begin{array}{cc} 0 & -22.5<\phi_{mn}^{c}\leq 22.5\\ 45 & 22.5<\phi_{mn}^{c}\leq 67.5\\ 90 & 67.5<\phi_{mn}^{c}\leq 112.5\\ 135 & 112.5<\phi_{mn}^{c}\leq 157.5\\ 180 & 157.5<\phi_{mn}^{c}\leq 202.5\\ 225 & 202.5<\phi_{mn}^{c}\leq 247.5\\ 270 & 247.5<\phi_{mn}^{c}\leq 292.5\\ 315 & 292.5<\phi_{mn}^{c}\leq 337.5 \end{array}\right.$$

Based on the above equation, only eight values of phases are considered during the simulation process, making the design less complex. Furthermore, the proposed quantized technique paves the way for later investigations regarding reconfigurable OAM-generating TAs using Varactor diodes or PIN diodes.

## 4. Experimental Verification

**Figure 4:**
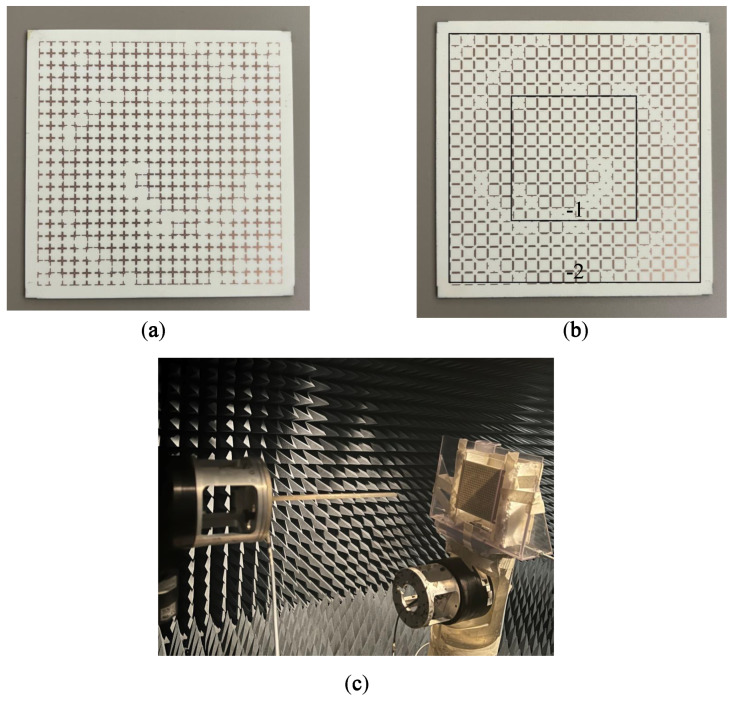
Configuration of the fabricated structure: (**a**) front side, and (**b**) back side.

The prototypes of the designed TA including 20 × 20 unit cells are fabricated on a Rogers 4003C with a thickness of 60 mils, as shown in Figure 4a,b. The centered TA with 10 × 10 unit cells helps to create OAM mode −1, and the rest of the unit cells are responsible for exciting OAM mode −2. In the figure, the mentioned sections are separated by square shapes.

**Figure 5:**
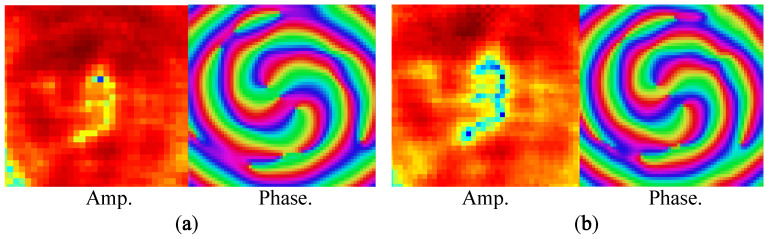
Amplitude and phase response for (**a**) X-polarization and (**b**) Y-polarization.

To verify the generation of mixed OAM modes, a setup as illustrated in Figure 4c was utilized. A probe scanning the square area of 15 × 15 cm ${}^{2}$ was placed 15 cm away from the proposed TA. The position of the TA (antenna under test) is fixed, while the probe (transmitter) scans the square area and transmits the signal each time. Thus, the amplitude and phase of the transmitted signal is detected by the TA at each position. Figure 5 depicts the phase front and amplitude response of the TA. As is evident, there is a null at the center of the scanning area, and two OAM modes (−1 and −2) are mixed together in the helical phase fronts.

**Figure 6:**
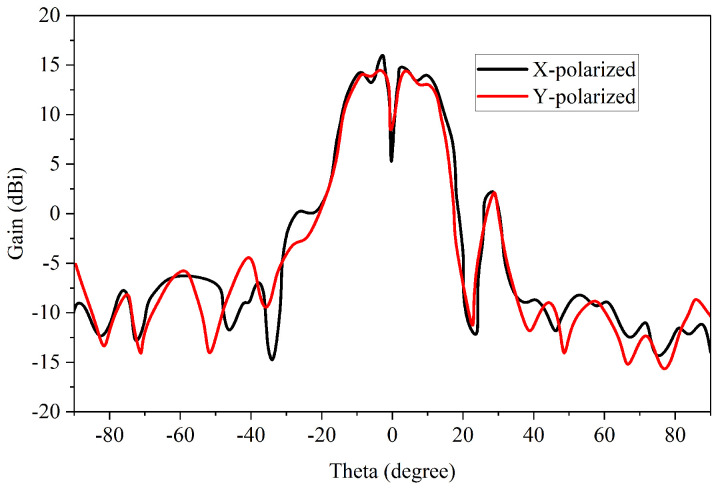
The far-field measured patterns for the suggested TA.

Concerning the far-field radiation pattern of the proposed TA, Figure 6 is provided. A maximum gain of around 16 dBi was achieved while the process was being measured, for both the X- and Y-polarization cases, confirming that the TA is dual-polarized. The existence of null at Theta = 0 is also obvious.

**Figure 7:**
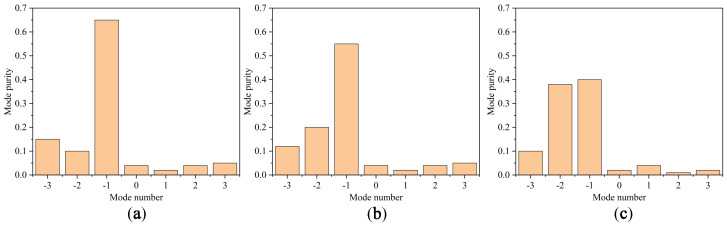
The mode purity analysis for X-polarization once (**a**) r = 4λ, (**b**) r = 8λ, and (**c**) r = 12λ.

Mode purity analysis is a factor which explores the validity of the generation of OAM modes. In a structure providing a single OAM mode, this value is 1, in theory. However, because the proposed TA excites two mixed OAM modes, the value depends on the circle in which the mode purity is calculated. In accordance with [59], the mode purity in both X- and Y-polarization was calculated. If we assign a circle with r = 4λ, taking into consideration the area inside the circle, a purity of around 0.65 was obtained for mode −1. As the circle becomes bigger, the purity of OAM mode −1 decreases, and that of −2 increases. Some results are presented in Figure 7. As the results for X- and Y-polarization cases were almost the same, we provide only the X-polarization scenario. It is worth pointing out that in an ideal situation, the mode purity should be 0.5 for modes −1 and −2. However, owing to fabrication and quantization errors, some impurities exist.

## 5. Discussion

As mentioned above, OAM antennas are mainly categorized into four groups. The first group is reconfigurable structures that generate one predefined mode at each time. More specifically, according to the state of PIN diodes or varactor diodes, the desired OAM mode is excited. This kind of antenna can be useful as a spatial filter. To demonstrate, if a mode-tunable antenna is utilized on a receiver side, it is possible to catch the signals that have the corresponding mode and refuse other unwanted modes. The second group is antenna arrays that have their own feeding networks. Each feed helps create the necessary phase distribution for one OAM mode. Due to the fact that every OAM mode has its own feeding structure, the complexity of the network increases. It should be pointed out that such antennas can be used in a case in which multiple streams of data are going to be sent at the same time and frequency. The data streams are not combined to each other because of the orthogonality of OAM modes. Concerning the third group, single antenna elements that can generate vortex beams have a low gain. Furthermore, these types of OAM antennas are able to produce two OAM modes at a specific frequency, limiting their application in high-data-rate wireless systems. As the fourth group of OAM antennas, transmit arrays and reflectarrays including various kinds of metamaterials excite only one OAM mode. In this case, increasing the channel capacity would not make sense, because it is not possible to send different versions of a signal or multiple data streams at the same time and frequency. To enable metamaterial-based OAM antennas to improve the channel capacity, one should provide mixed-OAM modes. In this manner, different versions of a signal are transmitted using different OAM modes, and consequently, the possibility of receiving a high-quality signal on the reception side increases. A few works have been carried out regarding this [52,53,54]. However, they suffer either from the complexity of fabrication or from not being dual-polarized structures. Moreover, none of them use quantized phase values during the design process. This feature tremendously simplifies the unit cell design process for TA.

Besides the TAs generating mixed OAM modes at the broadside, there exist some that can create a desired mode at an off-axis position [55,56]. By superimposing the phase values on the TA or reflectarray, it is possible to excite two OAM modes at two different positions. With regard to the law of reciprocity, in cases where the horn antennas are located at the off-axis position corresponding to the OAM mode direction, the mixed OAM modes propagate at the on-axis direction. Taking advantage of this feature, a multiplexing-demultiplexing link using two copies of the structure (for transmission and reception) is obtained. This type of structure cannot support the generation of more than two mixed OAM modes, as the technique of superimposing the phases on the TA deteriorates the purity of mode. However, in our structure, by increasing the sub-TAs, the number of mixed OAM modes can be easily increased, as reported in [52]. Furthermore, utilizing two feeding sources in applications requiring the reduction of the multipath fading effect makes the system complicated. Moreover, they do not provide dual-polarized characteristics as described in our work.

Concerning some applications for our structure, it is worth pointing out that our structure provides the generation of two mixed OAM modes. Because these two modes are orthogonal together and propagate simultaneously, two versions of the data stream are transmitted. These two versions are not combined, and consequently, the possibility of achieving a high-quality signal at the receiver side increases. This feature is similar to the MIMO application (multipath fading reduction). In line with the channel capacity for the MIMO structure, our structure also offers high-capacity communication. Another example of applications for the use of mixed OAM modes is imaging applications. In fact, more information from the object being imaged will be available using mixed OAM modes from a shared aperture. This could give rise to high-resolution imaging in forthcoming wireless systems.

To summarize the above discussion, it is important to clarify that the novelty of our structure is related to its being polarization independent. This is because of the fact that the unit cells are dual-polarized. Additionally, during the design process we used quantized values for the phases, which simplifies the configuration of the structure as well as its design complexity in comparison with similar works [52,53,54]. Further, this feature paves the way for introducing mode-tunable OAM-generating TAs, because creating quantized values by embedding active components such as pin diodes and varactor diodes is much easier than creating continuous phase values. Moreover, the fabrication cost of the suggested structure is low in comparison with those of the cited references, due to having ultrathin unit cells designed on a cheap substrate. Conversely, [52] requires state-of-the-art equipment for fabrication and [54] uses cascaded substrates. Among the applications for the proposed structure, point-to-point high-data-rate communications, high-resolution imaging, and high-quality sensing can be taken into consideration.

## 6. Conclusions

This study presented a dual-polarized transmit array (TA) that can generate mixed orbital angular momentum (OAM) modes. The proposed structure comprises two concentrically embedded TAs, where the inner TA has 10 × 10 Huygens’ metasurfaces to generate OAM mode −1, and the outer TA excites mode −2. The generation of OAM modes is achieved by adjusting the angular position of each unit cell and the necessary phase compensation for the conversion of the propagating spherical wave to a planar one. The results obtained demonstrate helical phase fronts composed of mixed −1 and −2 modes and radiation patterns that show null at the broadside. Maximum gains of 16 dBi were obtained in both X- and Y-polarization. Moreover, the mode purity analysis was evaluated, and the equality of mode −1 and −2 purity was confirmed, taking into account a larger calculating circle. Therefore, it is suggested that this proposed TA can be a potential candidate for enhancing the channel capacity of next-generation wireless communication systems and acquiring high-resolution images in imaging systems. 

## Data Availability

Not applicable.
